# From Prescription to Prevention: Patient Awareness, Risk Perceptions, and Predictors of Knowledge of Medication-Related Osteonecrosis of the Jaw

**DOI:** 10.3390/jcm15166280

**Published:** 2026-08-13

**Authors:** Gökçen Akçiçek, Münevver Gamze Diricanli, Nursel Akkaya

**Affiliations:** Department of Dentomaxillofacial Radiology, Faculty of Dentistry, Hacettepe University, Sıhhiye, 06100 Ankara, Türkiye; munevverdiricanli@hacettepe.edu.tr (M.G.D.); ynursel@hacettepe.edu.tr (N.A.)

**Keywords:** awareness, bisphosphonate-associated osteonecrosis of the jaw, drug-related side effects and adverse reactions, patient education

## Abstract

**Background/Objectives**: Proactively informing the patient prior to initiating medication and implementing preventive strategies are essential for mitigating the risk of developing medication-related osteonecrosis of the jaw (MRONJ). This study evaluated the awareness of MRONJ among a group of dental faculty patients who were scheduled to use, were using, or had previously utilized bisphosphonate and/or denosumab. **Methods**: A structured questionnaire-based survey was used as the survey tool to evaluate the patients’ awareness of MRONJ. A non-probability convenience sampling method was employed throughout a one-year period. **Results**: Throughout the study period, 202 participants were included, with a response rate of 97.58%. Only 35 (17.3%) of the patients had awareness of the risk of MRONJ, and 23 (11.4%) reported that their prescribing physicians had informed them about the potential side effects of their medication before the commencement of treatment. Univariate logistic regression, which was performed to elucidate the elements affecting awareness, revealed a significant odds ratio for healthcare professionals as a source of knowledge (29.229, *p* < 0.001), information of side effects given by a prescribing physician (25.343, *p* < 0.001), dental consultation (2.592, *p* = 0.018), and regular dental visits (3.205, *p* = 0.004). In the multivariate logistic regression analysis, “Healthcare professional as source of knowledge” was significantly associated with awareness (OR = 21.956, *p* < 0.001). “Information of side effects given by a prescribing physician” was also a significant predictor (OR = 11.450, *p* < 0.001), indicating that participants informed by prescribing physicians about the side effects were more likely to have awareness. **Conclusions**: This study revealed that despite patients’ limited awareness of MRONJ, the dissemination of information by healthcare professionals positively influenced their awareness and understanding of the risk factors associated with MRONJ. In particular, receiving information from healthcare professionals and prescribing physician-provided information about side effects appeared to be the strongest predictors of awareness.

## 1. Introduction

Medication-related osteonecrosis of the jaw (MRONJ), initially identified as bisphosphonate-related osteonecrosis of the jaw (BRONJ) in bisphosphonate (BP) recipients, is defined by the presence of exposed bone or bone reachable through probing via an intraoral or extraoral fistula in the maxillofacial area, persisting for over 8 weeks in patients with a history of treatment involving antiresorptive or antiangiogenic agents and in the absence of prior radiation therapy to the jaw or visible metastatic disease in the jaw [1,2,3]. The first case of BRONJ was documented in 2003, around 40 years after the medication’s introduction [4,5], and many cases have been reported since then [6,7,8]. Since 2005, the manufacturer has included this side effect in the package insert [9]. BPs are frequently utilized in the management of bone disorders, including osteopetrosis, osteopenia, Paget’s disease, and oncological conditions such as malignant hypercalcemia and multiple myeloma, as well as prostate and breast cancer [4,10]. In addition to BPs, various medications, including denosumab (receptor activator of nuclear factor kappa-beta ligand inhibitors), antiangiogenic agents, and recently tocilizumab (the humanized monoclonal antibody targeting the interleukin-6 receptor), which is utilized in inflammatory disorders such as rheumatoid arthritis and COVID-19, have been implicated in the etiology of MRONJ [1,2,3,11].

Although MRONJ can occur spontaneously, albeit infrequently, the likelihood of its development notably escalates following dental surgical operations that include bone manipulation, including tooth extractions. In addition, infectious and inflammatory dental diseases may contribute to its progression [1,3,12]. The risk rises in those who have utilized this medicine for over four years and in those concurrently consuming corticosteroids during that timeframe [2].

As it is challenging to manage MRONJ, and it markedly diminishes patients’ quality of life because of pain, infection, halitosis, and functional and aesthetic changes, preventing its development should be the primary goal [1,3,13,14]. Preventive dental care prior to treatment has been shown to reduce the occurrence of MRONJ [15]. Dental procedures, including tooth extraction, periodontal therapy, and endodontic treatment, should be conducted prior to the commencement of medication [14], and dentures must be examined to eliminate the possibility of their producing mucosal breaches [16]. Therefore, it is imperative to elucidate the prospective consequences to the patient prior to medication administration to guarantee the execution of the requisite dental treatments, to elevate oral hygiene to an ideal standard and to convey the significance of regular dental examinations as a component of preventative strategies [1,12,17]. For this reason, clinicians must possess an extensive awareness and understanding of MRONJ.

A multidisciplinary strategy for MRONJ prevention is recommended, which includes educating patients and healthcare professionals on the risks of MRONJ development, suitable preventative measures, and oral health guidance [13]. However, studies have shown low awareness among patients of MRONJ [14,18,19]. A study conducted in the UK showed that patients had insufficient information about what they should do to protect themselves from MRONJ [13]. Unfortunately, studies investigating MRONJ awareness among physicians and dentists also indicate that awareness levels are generally low; however, they differ by specialty, experience, and education [20,21,22,23,24]. A study investigating the awareness level of physicians prescribing the medication (denosumab or BP) revealed that 78.7% of physicians were aware of MRONJ; however, only 20.8% had good knowledge, 31.2–41.6% informed their patients about MRONJ, and only 20.8–25.3% referred patients to a dentist before starting the medication [22].

Proactively informing the patient prior to initiating medication and implementing preventive strategies are essential for mitigating the risk of developing MRONJ [22]. Although the patients perceived prescribers as playing the key role in articulating the risk of MRONJ [13], dentists must inform patients about their condition and the associated risks of dental operations. Consequently, it is important that prescribing physicians, irrespective of their specialty, and dentists, particularly, inform their patients.

Although, studies have shown low awareness among patients of MRONJ [14,18,19], research on the impact of several factors affecting this is insufficient. This study evaluated the awareness of MRONJ among a group of dental faculty patients who were scheduled to, were using, or had previously used antiresorptive/antiangiogenic medication, as well as the factors affecting this awareness.

## 2. Materials and Methods

Strengthening the Reporting of Observational Studies in Epidemiology (STROBE) Statement guidelines were adhered to in this study. A structured questionnaire-based survey was used as the survey tool to evaluate the patients’ awareness of MRONJ. The survey was adapted from previous studies [14,19,20,25,26] and conducted in Turkish. Four dentomaxillofacial radiologists and one endodontist evaluated the survey for its clarity and scope. After their feedback, modifications to the questionnaire were made, and the final questionnaire was obtained. The questionnaire began with a description, ensuring the participants’ voluntary participation and the anonymity of their responses. The survey form consisted of a total of 20 questions, including nine demographic questions, nine with yes/no responses, and two with multiple-choice responses (multiple different options that allowed for multiple responses). The detailed survey questionnaire is available in Appendix A.

This study was conducted at the Hacettepe University Faculty of Dentistry, Department of Dentomaxillofacial Radiology, and was reviewed and approved by the Non-Interventional Clinical Research Ethics Board of Hacettepe University (approval date: 1 November 2022, decision No.: 2022/18-16, project No.: GO 22/1115). All procedures were conducted in accordance with the principles of the Declaration of Helsinki. All patients who applied to a university hospital for various reasons and who were utilizing, had used, or intended to utilize antiresorptive/antiangiogenic medication between 1 November 2022 and 1 November 2023 were invited to join this questionnaire survey at their discretion to fill out the questionnaires without pressure from the investigators. Consequently, a non-probability convenience sampling method was employed to recruit accessible patients.

Following the acquisition of informed consent, the questionnaire forms were administered face-to-face by clinicians. Participation in the survey was entirely voluntary, and participants were informed that they could decline to participate or withdraw from the survey at any time without affecting their dental care. Participants were also informed that the collected data would be used solely for scientific purposes. The questionnaire took approximately 10 min to complete. During the survey process, the questions were posed verbally to the participants, and the answers were recorded by the physicians. The participants were allowed to ask questions, and any unclear items were explained by the clinicians to ensure accurate understanding. The collected data were recorded in an Excel spreadsheet and anonymized by assigning a unique identification number to each participant. No personally identifiable information, such as names or file numbers, was included in the dataset. The inclusion and exclusion criteria of this study are presented below.

Inclusion criteria:

- Individuals aged 18 and above;
- Current, past, or planned exposure to antiresorptive or antiangiogenic medications associated with MRONJ risk;
- Voluntary agreement to participate in this study.

Exclusion criteria:

- Refusal to participate in this study;
- Incomplete responses that precluded data analysis;
- Inability to understand and appropriately respond to the questionnaire.

### Statistical Analyses

The normality of the distribution of numeric variables was assessed using the Kolmogorov–Smirnov normality test for sample sizes of 50 or higher. The Shapiro–Wilk normality test was employed otherwise.

Associations between categorical variables were assessed using the Pearson chi-square test when the necessary conditions were satisfied. Alternatively, Fisher’s exact test or the Fisher–Freeman–Halton exact test was employed to assess the associations between categorical variables depending on the table size. Frequencies and percentages (n, %) are presented as descriptive statistics for categorical variables.

Univariate logistic regression analyses were first performed to identify factors associated with MRONJ awareness. Variables for inclusion in the analysis were initially obtained from questions structured based on clinical and conceptual relevance as identified in the established literature and then subjected to univariate statistical screening (*p* < 0.05) for the multivariable logistic regression model. The results of the regression analyses were reported as odds ratios (ORs) with 95% confidence intervals (95% CIs) and corresponding *p*-values.

The goodness of fit of the multivariable logistic regression model was assessed using the Hosmer–Lemeshow test. Model performance was further evaluated using the Cox & Snell R^2^ and Nagelkerke R^2^ statistics, as well as the overall classification accuracy, sensitivity, and specificity. Multicollinearity was assessed using adjusted generalized variance inflation factor (GVIF) values, with a threshold of adjusted GVIF < 2 considered acceptable.

Model discrimination was evaluated using the area under the receiver operating characteristic (ROC) curve (AUC) with DeLong 95% confidence intervals.

Internal validation was performed using bootstrap resampling with 2000 replications to assess model stability.

A two-sided *p*-value of <0.05 was considered statistically significant for all analyses. All analyses were performed using IBM SPSS Statistics for Windows, Version 23.0 (IBM Corp., released 2015, Armonk, NY, USA: IBM Corp.). The significance threshold was established at *p* < 0.05 for all analyses.

## 3. Results

Throughout the study period, 207 suitable patients were identified; five patients were excluded from this study as two refused to participate and three submitted incomplete questionnaires, resulting in a final study cohort of 202 participants and a response rate of 97.58%. The sample was predominantly female and had a median age of 65 years. The detailed demographic and baseline clinical characteristics of the participants are shown in Table 1.

Most of the participants (79.7%) had at least one systemic disease, with high blood pressure (46.5%), diabetes mellitus (22.8%), and thyroid diseases (19.8%) as the most common. Most individuals (n = 184, 91.1%) utilized the medicine for osteoporosis/osteopenia, with approximately half of the participants obtaining the medicine by parenteral administration. Among the 18 (8.9%) patients receiving antiresorptive/antiangiogenic therapy for oncological indications, the underlying diseases were multiple myeloma (n = 4), breast cancer with bone metastasis (n = 5), prostate cancer with bone metastasis (n = 3), lung cancer with bone metastasis (n = 1), and other malignant bone metastases (n = 5). Table 1 presents the comprehensive distributions of the administration routes, medication duration, and precise pharmacological classifications for both medical indications.

Only 35 (17.3%) of the patients were aware of the risk of MRONJ. Concerning proactive patient education, a comparably low percentage of participants (11.4%) reported that their prescribing physicians had informed them about the potential side effects of their medication before the commencement of treatment. Table 2 summarizes the comprehensive descriptive statistics, including the knowledge sources, perceptions of specific risk factors, and dental visit routines.

Bivariate analysis of the demographics demonstrated that sex was not significantly associated with most of the factors studied (*p* > 0.05), with a few exceptions. Male subjects had significantly higher frequencies of dental referrals before medication (46.2% vs. 18.2%, *p* = 0.001) and higher self-reported incidence of MRONJ development (15.4% vs. 3.4%, *p* = 0.027) compared with female subjects.

Age distribution was largely consistent across the variables, with the exception of dentists as a source of knowledge (*p* = 0.014), awareness of the risks associated with tooth extraction (*p* = 0.002), understanding the requirement of regular dentist visits (*p* = 0.044), and having information regarding side effects provided by prescribing physicians (*p* = 0.027). The average age of individuals in these groups was younger.

The relationship between education and variables was explored, and the educational level of the participants was significantly associated with awareness and behavioral parameters (Table 3). Higher educational backgrounds (bachelor’s degree or above) were associated with significantly higher rates of overall MRONJ awareness (27.5%) and were more likely to identify their medical doctors as their primary knowledge source (27.5%) compared with those with lower educational backgrounds (*p* < 0.05). Moreover, a better recognition of specific risk factors for MRONJ, such as tooth extraction (*p* = 0.002), dental infection (*p* = 0.001), and the possibility of spontaneous occurrence of necrosis (*p* = 0.032), showed a significant association with higher levels of education. Participants with higher education also showed a significantly higher adherence to regular dental visits after the start of the medication (*p* = 0.010). There was no significant association between educational status and other behavioral or referral parameters (*p* > 0.05).

Substantial variations were noted between the groups in the assessment of clinical indications for antiresorptive medication (Table 4). Patients undergoing treatment for oncological conditions were provided with proactive side-effect information by their prescribing physicians at a markedly higher rate than those treated for osteoporosis or osteopenia (*p* = 0.001). The incidence of MRONJ development was significantly higher in the cancer cohort compared with the osteoporosis or osteopenia cohort (*p* < 0.001).

The relationship between the method of medication administration and the onset of MRONJ was statistically significant (*p* < 0.001). None of the patients who administered the medicine parenterally had a history of MRONJ, while seven (11.5%) patients who administered the medication orally, and three (13.6%) who utilized the drug in combination had a prior history of MRONJ. There was also a statistically significant relationship between the medication administration route and the source of knowledge. The identification of dentists as a source of knowledge was more prevalent among individuals who administered medication parenterally compared with those who administered it orally and in combination, with frequencies of 17 (16.5%), 3 (4.9%), and 1 (4.5%), respectively (*p* = 0.044).

In this study, 68 patients (33.7%) indicated they had used the medicine for the last year, 50 patients (24.8%) reported usage for 1–5 years, and 12 patients (5.9%) stated they had used it for over 5 years. However, the length of pharmaceutical usage could not be accurately documented for 72 (35.6%) patients. Certain individuals in this cohort reported having previously utilized and discontinued the medicine but were unable to recall the duration, while others indicated they had used it, ceased, and resumed usage without recollection of their prior usage duration. Given the established correlation between medication use duration and MRONJ risk [1,2,3], these people were categorized as unknown and segregated from other groups in the assessments to prevent biasing the results. The correlations between the duration of pharmaceutical usage, awareness, information sources, and the risk of MRONJ are presented in Table 4. Although not statistically significant, there seems to be a correlation between increased awareness and the length of medication usage. There was a significant relationship between the duration of medication use and the source of information, the awareness that denture impaction constituted a risk factor, understanding the necessity for dental examinations, having information conveyed by the prescribing physician on side effects, and being referred for dental consultation.

Among the 23 patients who indicated that their prescribing physicians informed them about the medication’s side effects, 20 (87%) identified the medical doctor as the source of their information. A substantial correlation existed between prescribing physicians notifying patients about prescription side effects and their awareness, as well as their knowledge of the risk of tooth extraction, the risk of dental infection, denture sore risk, spontaneous development risk, and the necessity of attending regular dental check-ups (Table 4).

Among the 38 persons who identified their medical doctor as their source of knowledge, 21 (55.3%) were aware of MRONJ, whereas this awareness was shown in only 14 (8.5%) of the 164 patients who did not cite their medical doctor as their source. Individuals who identified the medical doctor as their source of information exhibited markedly elevated awareness, as well as knowledge of the risk of tooth extraction, the danger of dental infection, the risk of prosthetic injuries, the risk of spontaneous development, knowledge of dental checkup necessity, and the need for attendance at regular dental visits, and they were referred to dental consultations prior to medication (Table 5).

A total of 31 patients identified their dentist as their source of information, with 13 (41.9%) of them being aware. Individuals who identified their dentist as their source of information had markedly higher awareness, as well as knowledge of the risk of tooth extraction, and they were referred to dental consultations prior to medication (Table 5).

A higher greater percentage of individuals attending a dental consultation identified medical doctors and dentists as their sources of knowledge compared with those who did not attend a dental consultation (*p* = 0.013, *p* < 0.001). A notable correlation was identified between awareness, the knowledge of tooth extraction risk and dental infection risk, and the importance of regular dental examinations among individuals referred for dental consultation (Table 5).

After these results, regression analysis was performed to elucidate the elements affecting awareness.

The full model demonstrated good overall performance. The Hosmer–Lemeshow goodness-of-fit test was not statistically significant (χ^2^ = 1.797, df = 8, *p* = 0.987), indicating excellent agreement between the observed and predicted outcomes. The model explained a moderate-to-high proportion of the variance in awareness, with a Cox & Snell R^2^ of 0.334 and a Nagelkerke R^2^ of 0.554. The overall classification accuracy of the model was 88.6%. The model showed high specificity (95.2%), correctly identifying participants without awareness, while the sensitivity was lower (57.1%), indicating a more limited ability to correctly identify participants with awareness.

Univariate logistic regression revealed a significant odds ratio (OR) (13.235, *p* < 0.001) for medical doctors as a source of knowledge, for dentists as a source of knowledge (4.891, *p* < 0.001), healthcare professionals as a source of knowledge (29.229, *p* < 0.001), information of side effects given by a prescribing physician (25.343, *p* < 0.001), dental consultation (2.592, *p* = 0.018), and regular dental visits (3.205, *p* = 0.004). For education, the “High school or below” category showed borderline significance (0.274, *p* = 0.003), suggesting that a lower educational level may be associated with lower awareness (Table 6).

The source of knowledge variable was assessed as a multiple-response item, allowing participants to select more than one information source. Consequently, the response categories were not mutually exclusive. To account for this structure, each source of knowledge category was transformed into a separate dichotomous (yes/no) variable and considered independently for the multivariate logistic regression model.

In the multivariate logistic regression analysis, “Healthcare professional as source of knowledge” was significantly associated with awareness (OR = 21.956, *p* < 0.001). Participants who received information from healthcare professionals had higher awareness levels. “Information of side effects given by a prescribing physician” was also a significant predictor (OR = 11.450, *p* < 0.001), indicating that participants informed by prescribing physicians about side effects were more likely to have awareness (Table 6).

Age showed borderline significance in the multivariate logistic regression analysis (OR = 1.042, *p* = 0.067), suggesting a slight increase in awareness with increasing age.

Although education demonstrated a significant association in the univariate analysis (*p* = 0.003), it did not retain statistical significance in the multivariable model (overall *p* = 0.111), being above the conventional threshold of 0.05 for statistical significance. This attenuation is likely attributable to the limited sample size, particularly the small no-education subgroup (n = 26, 12.9%), which reduced the statistical power to detect an independent effect after adjusting for other predictors. Nevertheless, the effect estimates for each education category (no education: OR = 0.176, 95% CI: 0.023–1.374; high school or below: OR = 0.349, 95% CI: 0.101–1.204) are provided alongside their confidence intervals; sex, regular dental visits, and dental consultation were also not significant in the multivariable analysis. Multicollinearity diagnostics revealed adjusted GVIF values ranging from 1.053 to 1.167, well below the conventional threshold of 2.0, indicating no substantial collinearity between the predictors.

The model demonstrated excellent discriminative ability with an AUC of 0.925 (95% CI: 0.883–0.967; DeLong method).

Bootstrap internal validation with 2000 replications yielded a mean optimism of 0.029 and an optimism-corrected AUC of 0.896 (C-Index), indicating model stability and minimal overfitting.

## 4. Discussion

Multiple factors influence the onset of MRONJ, including pharmaceutical type, route of administration, indication for use, duration of use, and any coexisting systemic disorders and medications [1,3,12,27]. Based on these factors, the likelihood of developing MRONJ varies from 0.15% to 14.8% [1], and its incidence ranges from 0% to 3.2% [12]. Antiresorptive agents such as BPs and denosumab exhibit a heightened susceptibility in patients to the onset of MRONJ. Furthermore, the probability of developing MRONJ is higher in those with malignancies (<5%) than in those with osteoporosis (<0.05%) [1]. In this study, among the 202 patients, 5% had a history of MRONJ, and it developed more frequently in individuals using medication for oncological reasons, which is consistent with the literature [12,27]. The elevated incidence of MRONJ in this study compared with the literature may be attributed to the inclusion of only those patients who presented at the university hospital and were currently using or had previously used such medication. In addition, this study was single-center and used convenience sampling, which may have introduced selection bias (e.g., patients who are more health-conscious may be more likely to attend a university hospital). However, considering the increased number of systemic diseases requiring antiresorptive agents and/or other medications [12], the growing elderly population globally [28], and the consequent escalation in medication usage, especially concerning osteoporosis, it is foreseeable that cases of MRONJ will increase in the future.

The occurrence of MRONJ is significantly reduced in patients employing preventive measures, including patient education and daily oral hygiene [29,30]. Multiple studies and position papers in the literature underscore the necessity of informing patients about the potential side effects prior to initiating this medication, as well as the importance of referring them to a dentist and addressing any existing dental infections to avert the onset of MRONJ [1,29].

This study evaluated the awareness of MRONJ among patients who applied to a university hospital for various reasons and who had used, were utilizing or intending to utilize BP and/or denosumab. Among the 202 patients involved in the trial, only 35 (17.3%) exhibited awareness. This low awareness level was similar to that of other studies [14,17,18,19,25,31]. Abdullateef and Alhareky [18] reported poor awareness (33.82%) among dental patients now or potentially receiving therapy with BP, denosumab, and antiangiogenic medicines. El-Ma’aita et al. [19] evaluated the awareness of the risk of developing MRONJ among BP users and found low (12.4%) awareness. They reported that only five (33%) of these patients were informed by their prescribing physicians, six (40%) by their dentists, and seven (26.7%) by other sources and self-education [19]. In a separate study conducted by Bauer et al. [14], 62% of patients reported obtaining information from the drug’s package insert and 29% from the physicians. In the study by Migliorati et al. [26], 18.2% of patients said that their prescribing physicians told them about the risks associated with the medication. In this study, 23 participants (11.4%) were informed by their prescribing physicians about the potential adverse effects of the medication prior to its prescription. Conversely, as the source of information, 38 (18.8%) individuals reported their medical doctor, 31 (15.3%) their dentists, and 17 (8.4%) from alternative sources. In a systemic review of six studies with 483 patients included, regarding the source of information, package inserts and health specialists were the common sources [17]. The information sources in this research did not include package inserts, potentially due to the dissimilarity of the patient population with other studies.

In the present study, to enhance comprehension of the elements influencing awareness, regression analyses were conducted, and the results showed that individuals whose information source was a healthcare professional had a probability of approximately 22-times-higher awareness levels, and those informed about the drug’s side effects by the prescribing physician had a probability of approximately 11-times-higher awareness levels. These results clearly illustrate the influence of healthcare practitioners disseminating information to patients, whether during the prescription phase or subsequently, on their level of MRONJ awareness.

As MRONJ is a disorder resulting from pharmaceutical usage, the prescribing physician is the primary individual responsible for informing the patient [32]. Similarly, patients perceived prescribers as playing the key role in articulating the risk of MRONJ [13]. However, as dental surgical procedures such as tooth extraction are among the risk factors, dentists should also inform these patients prior to the dental treatments, and effective interprofessional patient education and prevention of the development of MRONJ are required [13,32]. Al-Samman’s 2019 [21] study assessed the awareness and knowledge levels of 178 dentists, revealing that awareness was 33.6% among general dental practitioners, 48.5% among dental radiologists, and 84.4% among maxillofacial surgeons. In a multicenter study, awareness among dentists was 83.3% [33]. Enhancing patients’ awareness and understanding is essential for the prevention of MRONJ. Dentists, pharmacists, and family physicians can all furnish this information [19,34]. The patients’ limited knowledge and awareness may stem from the insufficient knowledge and awareness of their physicians [14,19] or from the failure to communicate this information to the patients [22]. Research indicates that physicians possess a commendable level of awareness and expertise concerning MRONJ; however, they inadequately convey this information to their patients [22,35]. Physicians must apprise their patients of the possible side effects of the medicine and the significance of dental therapies to mitigate the risk of MRONJ [32]. For this reason, it has been proposed that written instructions be supplied to patients [36]. In Türkiye, prescriptions are issued via a digital system associated with the Ministry of Health, and patients receive notifications of their prescriptions through texts sent to their mobile devices. An alternative option would be to create additional software capable of transmitting a guidance message to the patient regarding the adverse effects of the medication and preventive measures, alongside the prescription notification for drugs that may induce MRONJ. Moreover, the authors plan to provide patients with written informational brochures alongside their prescriptions, especially targeting the elderly and individuals less familiar with technology, as indicated by the existing literature [36].

Osteoporosis was the predominant indication for pharmacological intervention, consistent with the literature [17,26,31]. Unfortunately, in the osteoporosis/osteopenia group prescribing physicians provided less information regarding side effects (8.7%) and referrals for dental consultations (20.7%) compared with cancer patients (38.9% and 33.3%, respectively). Similarly, among the patients using medication for osteoporosis/osteopenia, fewer (16.8%) stated the medical doctor as their source of knowledge, compared with patients using medication for oncological reasons (38.9%). While this difference was not statistically significant, it was clinically important. This discrepancy may be interpreted as physicians providing oncology patients with additional information regarding the medication and its adverse effects or as patients adopting a more serious approach to the problem. MRONJ risk factors such as tooth extraction, dental infection, denture soreness, and spontaneous development were better known in oncology patients, but no significant difference was observed between the two groups. Further studies with more individuals are needed to better understand these relationships.

In addition, while only 15.8% of the osteoporosis/osteopenia patients were aware of MRONJ, 33.3% of the oncology patients were aware. Bauer et al. [14] indicated that nearly half of cancer patients had supplementary information from an oncologist, whereas just 17% of osteoporosis patients received additional information from a specialist. Healthcare personnel assisting cancer patients typically possess a superior understanding of the dentist’s role in the prevention of MRONJ [24] and are more inclined toward dental referrals [23]. Although not statistically significant in this study, patients using medication for oncological reasons demonstrated a higher likelihood of being referred for dental consultations. The current study revealed a similar rate of referral for dental consultation (21.8%) to the literature [22,23,35]; however, there are lower [19,31] and higher [18] referral rates. Kim et al. [23] reported that the primary reasons for refraining from dental referrals are that medical doctors often do not perceive a necessity for dental consultation; they encounter challenges in collaborating with patients due to financial, temporal, and motivational constraints; and particularly in oncological cases, the management of systemic disease takes priority over dental care. In this study, there was a statistically significant relationship between individuals referred for dental consultation and their source of knowledge and awareness. Although the dental consultation showed a significant association, with referred patients having approximately 2.6-times-higher odds of awareness in univariate logistic regression analyses, in the multivariate logistic regression analyses, this relationship was not significant, suggesting that the apparent univariate association was confounded by other variables, particularly healthcare professional information sources. The rationale for these associations may stem from the information provided to patients by their physicians during consultations, as well as insights acquired from dentists during dental examinations. Consequently, it is essential for prescribing physicians, without regard to the medication indication, to educate these patients and refer them for dental consultation to ensure dental treatments and the maintenance of optimal oral hygiene prior to the initiation of the medication. In the event that a standardized referral form or digital notification system is available, physicians who prescribe antiresorptive/antiangiogenic medication may utilize it to refer patients for dental consultations.

The present study’s findings indicated a low awareness of MRONJ risk factors such as dental surgical operations, including tooth extractions (16.3%); dental infections (9.4%); oral mucosal injuries, such as denture impaction (5%); and spontaneous occurrence (6.9%). Studies demonstrate that healthcare providers are also unaware of the MRONJ risk factors. In the study by Miranda-Silva et al. [24], among 1370 health professionals, awareness of risk factors such as trauma and the use of ill-fitting dentures was 35.95%, 17.99%, and 40.40% among dentists, physicians, and nurses, respectively. The risk factors, causal medications, and therapeutic methods associated with MRONJ are perpetually being revised. Therefore, it is crucial for healthcare professionals to be informed of this contemporary knowledge via postgraduate training to enhance awareness and prevent the onset of MRONJ through protective strategies.

Awareness was affected by gender, education level, and the drug’s administration technique in some studies [18,19,25]. In the present study, gender and the drug’s administration technique had no effect on awareness, but it was associated with the education level. Bivariate analysis showed that higher educational backgrounds (bachelor’s degree or above) were associated with significantly higher rates of overall MRONJ awareness (27.5%, *p* = 0.003). Similarly, the univariable regression analysis showed significance (0.274, *p* = 0.003), suggesting that a lower educational level may be associated with lower awareness. However, it was not statistically significant (*p* = 0.111) in the multivariable analysis. There were only 26 (12.9) patients in the no-education group, and this could have affected the results. Examining larger samples may enhance the understanding of the influence of education level.

There seems to be a correlation between increased awareness and the length of medication usage; however, this was not statistically significant. There was a significant relationship between the duration of medication use and the source of information (medical doctor *p* = 0.022, dentist *p* = 0.011), understanding the necessity for dental examinations (*p* = 0.020), information conveyed by the prescribing physician on side effects (*p* = 0.018), and referral for dental consultation (*p* < 0.001). These findings may be attributed to patients who had been utilizing the medicine for an extended duration, resulting in more frequent meetings with their physicians and a superior retention of repeated information. Likewise, individuals who had utilized the medicine for an extended duration may have obtained additional information from dentists due to their increased frequency of dental visits during this time. The elevated rate among individuals previously unacquainted with the medicine may be attributable to this. Although the precise timeframe remains uncertain, considering their historical usage patterns and the probability of dental visits during this period, it is plausible that they received recurring information from dentists. The elevated frequency of dental visits among individuals previously unacquainted with the medicine may also stem from the fact that these patients had previously utilized the medication and intended to use it again.

Coexisting systemic disorders, including diabetes mellitus, anemia, hypertension, and rheumatoid arthritis, and medications such as corticosteroids and chemotherapy are risk factors for the development of MRONJ [1,12]. In the current analysis of 202 patients, 161 (79.7%) exhibited at least one concomitant systemic condition, and among the 10 patients with prior MRONJ history, nine had coexisting systemic disorders, including diabetes mellitus, anemia, hypertension, and arteriosclerosis.

Regular dental appointments are crucial for sustaining optimal oral health for the prevention of MRONJ. Regular dental examinations facilitate the early detection of caries and periodontal problems, preventing the need for invasive treatments such as tooth extraction or surgical interventions. In this study, 22 (10.9%) patients were aware of the need for regular dental exams after commencing the medicine to reduce the risk of osteonecrosis, and 39 (19.3%) were attending regular dental examinations after initiating the medication. These results were lower than those of the other studies [31]. The explanation for this may stem from the inadequate awareness of the study participants and possibly the differences in the patient demographics relative to prior research. Although the univariate logistic regression indicated a significant OR for regular dental visits (3.205, *p* = 0.004) regarding awareness, in the multivariate logistic regression, this connection was found to be insignificant (OR = 0.966, *p* = 0.956). Further studies including a larger cohort from diverse centers are necessary to elucidate this association more completely.

In this study, patients only reported BP and denosumab as medication. Although these are the most commonly associated medications with MRONJ, there are more recently reported medications, such as anti-TNF agents [37], tocilizumab [11], and tyrosine kinase inhibitors [38]. Future studies could focus on awareness of these medications among healthcare professionals and patients.

The main limitation of this study is that it includes patients from a single institution only. This study was conducted at a university hospital with established consultation connections between medical and dental professionals; therefore, the general awareness and attitudes of the participating patients may not be generalized to the broader public. Studies with larger sample sizes from many sites with adequate power might improve the understanding of the level of awareness and the factors impacting it, as this study used convenience sampling, which may have introduced selection bias (e.g., patients who are more health-conscious may be more likely to attend a university hospital). The information gathered in this research relies on questionnaires, which are derived from the participants’ responses. This situation could be prone to recall bias, especially among individuals who struggle to recollect the length of their previous medication, as patients with heightened awareness might be more inclined to remember or disclose having received information. Furthermore, the patients’ awareness of the adverse effects of medications was not studied, and a further study can be undertaken to assess their knowledge of these effects. In this study, a structured questionnaire-based survey adapted from previous studies [14,19,20,25,26] was used as the survey tool to evaluate patients’ awareness of MRONJ. Although the survey was evaluated by specialists regarding its clarity and scope, validation was not performed. To better understand patients’ awareness of MRONJ and its risk factors, the validity of the survey may be assessed in further research, and researchers could use psychometrically validated questionnaires to allow for better cross-study comparison of MRONJ awareness levels.

## 5. Conclusions

This study revealed that despite patients’ limited awareness (17.3%) of MRONJ, the dissemination of information by healthcare professionals positively influenced their awareness and understanding of the risk factors associated with MRONJ. In particular, receiving information from healthcare professionals and prescribing-physician-provided information about side effects appeared to be the strongest predictors of awareness. A systematic and organized interdisciplinary strategy requiring the prescribing physician documenting a mandatory dental clearance before the initial dose administration will significantly address this issue. Preventing this condition, which impairs a patient’s quality of life and is challenging to cure, is highly significant. Consequently, enhancing patients’ awareness and knowledge of this issue by healthcare providers is a crucial measure to mitigate MRONJ cases.

## Figures and Tables

**Table 1:** Descriptive characteristics of the participants (n = 202).

Variable	Descriptive Statistics n (%)
Age *	65 (59–70)
Sex (Female)	176 (87.1)
Education	
No education	26 (12.9)
High school or below	85 (42.1)
Bachelor or above	91 (45.0)
Systemic Disease	161 (79.7)
Diabetes mellitus	46 (22.8)
Hypertension/CVDs	94 (46.5)
Rheumatoid arthritis	25 (12.4)
Renal failure	1 (0.5)
Thyroid disease	40 (19.8)
Malignancy	36 (17.8)
Other systemic diseases	66 (32.7)
Steroid/Immunosuppressive Use	7 (3.5)
Medication Group (Type)	
BP	139 (68.8)
Denosumab	48 (23.8)
Combined	5 (2.5)
Unknown	10 (5.0)
Administration Route	
Oral	61 (30.2)
Parenteral	103 (51.0)
Combined	22 (10.9)
Unknown	16 (7.9)
Medication Indication	
Osteoporosis/osteopenia	184 (91.1)
Oncological	18 (8.9)
Usage Status	
Past use	45 (22.3)
Currently using	127 (62.9)
Planning	11 (5.4)
Other	19 (9.4)
Duration	
Last year	68 (33.7)
1–5 years	50 (24.8)
≥5 years	12 (5.9)
Unknown	72 (35.6)

* Median (IQR: P25-P75). CVDs: cardiovascular diseases.

**Table 2:** Awareness and knowledge about MRONJ.

Variable	Descriptive Statistics n (%)
Awareness	35 (17.3)
Knowledge Source	
Medical doctor	38 (18.8)
Dentist	31 (15.3)
Other	17 (8.4)
Risk Awareness	
Dental surgical operations (tooth extraction)	33 (16.3)
Dental Infection	19 (9.4)
Oral mucosal injuries (denture impaction)	10 (5.0)
Spontaneous risk awareness	14 (6.9)
Knowledge of regular dental check-up necessity	22 (10.9)
Attending regular dental visits	39 (19.3)
Informed about side effects by prescribing physician	23 (11.4)
Dental consultation prior to medication	44 (21.8)
Occurrence of necrosis	10 (5.0)
Reason for necrosis	
Extraction	8 (4.0)
Dental infection	1 (0.5)
Other	1 (0.5)

**Table 3:** The relation between education and variables.

	Education			
	No Education n (%)	High School or Below n (%)	Bachelor or Above n (%)	*p*
Awareness	2 (7.7)	8 (9.4)	25 (27.5)	0.003 *
Source of knowledge: MD	3 (11.5)	10 (11.8)	25 (27.5)	0.019
Risk of tooth extraction	3 (11.5)	6 (7.1)	24 (26.4)	0.002 *
Risk of dental infection	1 (3.8)	2 (2.4)	16 (17.6)	0.001 *
Risk of spontaneous occurrence	1 (3.8)	2 (2.4)	11 (12.1)	0.032 *
Attending regular dental visits	3 (11.5)	10 (11.8)	26 (28.6)	0.010 *

* Pearson’s chi-square *p* values were given. Otherwise, the Fisher–Freeman–Halton exact test was used. MD: medical doctor.

**Table 4:** The correlations among the drug indication, duration of pharmaceutical usage, information of side effects given by the prescribing physician and the variables.

Variable	Drug Indication			Duration					ISEGPP		
	Osteoporosis n (%)	Oncological n (%)	*p*	Last Year n (%)	1–5 Years n (%)	≥5 Years n (%)	Unknown n (%)	*p*	No (n, %), n = 179	Yes (n, %) n = 23	*p*
Awareness	29 (15.8)	6 (33.3)	0.095	8 (11.8)	8 (16.0)	4 (33.3)	15 (20.8)	0.217	18 (10.1)	17 (73.9.)	<0.001
Source of knowledge: MD	31 (16.8)	7 (38.9)	0.051	6 (8.8)	12 (24.0)	5 (41.7)	15 (20.8)	0.022 *	18 (10.1)	20 (87.0)	<0.001
Source of knowledge: dentist	28 (15.2)	3 (16.7)	0.743	6 (8.8)	4 (8.0)	2 (16.7)	19 (26.4)	0.011 *	28 (15.6)	3 (13.0)	1.000
Source of knowledge: other	16 (8.7)	1 (5.6)	1.000	9 (13.2)	3 (6.0)	1 (8.3)	4 (5.6)	0.349	14 (7.8)	3 (13.0)	0.419
Risk of tooth extraction	27 (14.7)	6 (33.3)	0.086	6 (8.8)	8 (16.0)	4 (33.3)	15 (20.8)	0.093 *	17 (9.5)	16 (69.6)	<0.001
Risk of dental infection	15 (8.2)	4 (22.2)	0.073	4 (5.9)	6 (12.0)	3 (25.0)	6 (8.3)	0.172	7 (3.9)	12 (52.2)	<0.001
Risk of denture impaction	8 (4.3)	2 (11.1)	0.220	2 (2.9)	3 (6.0)	3 (25.0)	2 (2.8)	0.031	2 (1.1)	8 (34.8)	<0.001
Spontaneous risk	11 (6.0)	3 (16.7)	0.116	3 (4.4)	2 (4.0)	3 (25.0)	6 (8.3)	0.088	4 (2.2)	10 (43.5)	<0.001
Knowledge of dental check-up	19 (10.3)	3 (16.7)	0.423	4 (5.9)	8 (16.0)	4 (33.3)	6 (8.3)	0.020 *	8 (4.5)	14 (60.9)	<0.001
Attending regular dental visits	35 (19.0)	4 (22.2)	0.756	12 (17.6)	11 (22.0)	3 (25.0)	13 (18.1)	0.879 *	28 (15.6)	11 (47.8)	0.001
The prescribing physician informed side effects	16 (8.7)	7 (38.9)	0.001	3 (4.4)	8 (16.0)	4 (33.3)	8 (11.1)	0.018 *	-	-	-
Dental consultation prior to medication	38 (20.7)	6 (33.3)	0.234	5 (7.4)	8 (16.0)	2 (16.7)	29 (40.3)	<0.001 *	-	-	-
Occurrence of necrosis	4 (2.2)	6 (33.3)	<0.001	3 (4.4)	3 (6.0)	0 (0.0)	4 (5.6)	0.959	7 (3.9)	3 (13.0)	0.091

All the *p*-values are related to Fisher’s exact test except * (Pearson’s chi-square test). MD: medical doctor; ISEGPP: information of side effects given by the prescribing physician.

**Table 5:** The correlations among dental consultation, source of knowledge and the variables.

Variable	Dental Consultation			Source of Knowledge					
	No n = 158 n (%)	Yes n = 44 n (%)	*p*	MD n (%)	*p*	Dentist n (%)	*p*	Other Sources n (%)	*p*
Source of knowledge: MD	24 (15.2)	14 (31.8)	0.013 *	-	-	-	-	-	-
Source of knowledge: dentist	14 (8.9)	17 (38.6)	<0.001 *	-	-	-	-	-	--
Source of knowledge: other sources	17 (10.8)	0 (0)	0.027	-	-	-	-	-	-
Awareness	22 (13.9)	13 (29.5)	0.015 *	21 (55.3)	<0.001 *	13 (41.9)	<0.001 *	9 (52.9)	<0.001
Risk of tooth extraction	21 (13.3)	12 (27.3)	0.027 *	19 (50.0)	<0.001 *	14 (45.2)	<0.001 *	6 (35.3)	0.039
Risk of dental infection	11 (7.0)	8 (18.2)	0.038	14 (36.8)	<0.001	6 (19.4)	0.086	5 (29.4)	0.013
Risk of denture impaction	6 (3.8)	4 (9.1)	0.229	9 (23.7)	<0.001	2 (6.5)	0.653	3 (17.6)	0.041
Spontaneous risk	8 (5.1)	6 (13.6)	0.085	11 (28.9)	<0.001	4 (12.9)	0.238	3 (17.6)	0.101
Knowledge of dental check-up	12 (7.6)	10 (22.7)	0.011	15 (39.5)	<0.001	5 (16.1)	0.345	4 (23.5)	0.097
Regular dental visits	29 (18.4)	10 (22.7)	0.516 *	16 (42.1)	<0.001 *	6 (19.4)	1.000	5 (29.4)	0.332
Occurrence of necrosis	8 (5.1)	2 (4.5)	1.000	2 (5.3)	1.000	1 (3.2)	1.000	0 (0.0)	1.000
The physician informed side effects	-	-	-	20 (52.6)	<0.001	3 (9.7)	1.000	3 (17.6)	0.419
Dental consultation prior to medication	-	-	-	14 (36.8)	0.013 *	17 (54.8)	<0.001 *	0 (0.0)	0.027

All the *p*-values are related to Fisher’s exact test except * (Pearson’s chi-square test). MD: medical doctor.

**Table 6:** Regression analysis of the elements affecting awareness.

Variable	Univariate OR (95% CI)	*p*	Multivariate OR (95% CI)	*p*
Age	0.992 (0.964–1.021)	0.585	1.042 (0.997–1.090)	0.067
Sex				
Male	1.159 (0.405–3.316)	0.784	0.539 (0.120–2.411)	0.418
Education		0.004		0.111
No education	0.220 (0.048–1.000)	0.050	0.176 (0.023–1.374)	0.098
High school or below	0.274 (0.116–0.649)	0.003	0.349 (0.101–1.204)	0.096
Source of knowledge				
MD	13.235 (5.703–30.718)	<0.001	—	—
Dentist	4.891 (2.107–11.355)	<0.001	—	—
Other sources	6.880 (2.435–19.439)	<0.001	—	—
Healthcare professional as source of knowledge	29.229 (9.672–88.332)	<0.001	21.956 (6.083–79.243)	<0.001
Information of side effects given by prescribing physician	25.343 (8.864–72.454)	<0.001	11.450 (2.955–44.369)	<0.001
Dental consultation prior to medication	2.592 (1.178–5.705)	0.018	0.606 (0.176–2.086)	0.427
Attending regular dental visits	3.205 (1.435–7.155)	0.004	0.966 (0.283–3.294)	0.956

MD: medical doctor; OR: odds ratio.

## Data Availability

The data presented in this study are available on request from the corresponding author.

## Abbreviations

The following abbreviations are used in this manuscript:

AUC	Area under the ROC curve
BP	Bisphosphonate
BRONJ	Bisphosphonate-related osteonecrosis of the jaw
CIs	Confidence intervals
COVID-19	Coronavirus disease 2019
CVD	Cardiovascular diseases
GVIF	Generalized variance inflation factor
ISEGPP	Information of side effects given by the prescribing physician
MD	Medical doctor
MRONJ	Medication-related osteonecrosis of the jaws
OR	Odds ratio
ROC	Receiver operating characteristic
STROBE	Strengthening the Reporting of Observational Studies in Epidemiology
UK	United Kingdom

## Appendix A. MRONJ Awareness Questionnaire

Demographic Findings:

1. Age:
2. Sex: F/M
3. Education:
4. Systemic Diseases:
5. Medication:
  - BP
  - Denosumab
  - Other
6. Medication indication:
  - Osteoporosis/Osteopenia
  - Malignancy
  - Other
7. Administration Route:
  - Parenteral
  - Oral
  - Unknown
8. How long have you been using the medicine?
9. Have you previously utilized these medications? If so, for what duration, with which administration technique, and when was the treatment terminated?
  - Duration:
  - Administration technique:
    Parenteral
    Oral
    Unknown
  - Time of treatment termination:

Awareness:

1. Are you aware that the use of this medication carries a risk of osteonecrosis, characterized by necrotic bone regions in the jaw, resulting from drug-induced alterations in the jawbone?
  Yes/No
2. From where did you obtain the information on the drug’s side effects?
  - Medical doctor
  - Dentist
  - Internet
  - Drug packages insert
  - Other
  - I don’t know
3. Are you aware that any surgical operation, including tooth extraction, poses a risk of osteonecrosis (necrotic bone regions in the jawbone)?
  Yes/No
4. Are you aware of the potential risk of osteonecrosis (necrotic bone regions in the jaw) resulting from inflammation/infection that may arise in your teeth?
  Yes/No
5. Are you aware that there is a risk of developing osteonecrosis (necrotic bone regions in the jaw) following oral mucosal injuries, such as those induced by removable dentures?
  Yes/No
6. Are you aware that the absence of dental procedures (spontaneous) still poses a danger of osteonecrosis (necrotic regions in the jawbone)?
  Yes/No
7. Are you aware that regular dental exams are necessary after commencing the medicine to reduce the risk of osteonecrosis?
  Yes/No
8. To minimize the risk of developing osteonecrosis, do you attend regular dental examinations after initiating the medication?
  Yes/No
9. Did your prescribing physician notify you of the potential negative effects of the medicine prior to its prescription?
  Yes/No
10. Were you referred to a dentist for a consultation about your oral health prior to commencing medication?
  Yes/No
11. Have you previously developed osteonecrosis in your jawbones?
  Yes/No
  Is the cause for its development known?
  - After radiotherapy
  - Tooth extraction
  - Dental inflammation
  - Mucosal injuries induced by removable dentures
  - Other:
